# Orthotopic hepatic cancer: radiofrequency hyperthermia-enhanced intratumoral herpes simplex virus-thymidine kinase gene therapy

**DOI:** 10.18632/oncotarget.23586

**Published:** 2017-12-22

**Authors:** Fu Xiong, Feng Zhang, Yin Jin, Qiaoyou Weng, Jingjing Song, Guofeng Zhou, David Shin, Chuansheng Zheng, Xiaoming Yang

**Affiliations:** ^1^ Department of Radiology, Union Hospital, Tongji Medical College, Huazhong University of Science and Technology, Wuhan, Hubei Province 430022, China; ^2^ Image-Guided Bio-Molecular Intervention Research and Section of Vascular & Interventional Radiology, Department of Radiology, University of Washington School of Medicine, Seattle, WA 98109, USA

**Keywords:** radiofrequency hyperthermia, gene therapy, hepatocellular carcinoma, molecular imaging

## Abstract

**Purpose:**

To validate the feasibility of using interventional radiofrequency hyperthermia(RFH) to enhance herpes simplex virus-thymidine kinase (HSV-TK)/ganciclovir (GCV) gene therapy of rat orthotopic hepatic cancer.

**Material and Methods:**

Rat hepatocellular carcinoma cells (MCA-RH-7777) were transduced with lentivirus/luciferase gene for optical imaging. *In-vitro* experiments with the luciferase cells and *in-vivo* experiments on rats with orthotopic hepatic tumors were divided into four treatment groups: (i) HSV-TK/GCV-mediated gene therapy combined with RFH; (ii) gene therapy alone; (iii) RFH alone; and (iv) phosphate buffered saline (PBS). Cell viability was evaluated by MTS assay and confocal microscopy, and HSV-TK gene expression in cells and tumors was quantified by western blotting. Bioluminescent optical imaging and ultrasound imaging were used to monitor and compare the photon signal and tumor size changes among different treatment groups overtime, respectively. The imaging findings were correlated with histology.

**Results:**

For *in-vitro* experiments, the combination therapy group (gene therapy + RFH) demonstrated the lowest cell proliferation by MTS assay, compared to the gene therapy alone, RFH alone, and PBS (26.1±3.2% vs 50.4±4.6% vs 82.9±6.3% vs 100%, p<0.01). The combination therapy group also showed fewer survived cells by the confocal microscopy and the lowest bioluminescent signal by the optical imaging. For *in-vivo* experiments, the combination therapy group demonstrated a significantly decreased signal intensity on the bioluminescent optical imaging (0.57±0.09, 1.06±0.10 vs 3.43±0.27 vs 3.85±0.12, p<0.05) and smallest tumor volume by ultrasound imaging (0.28±0.11 vs 1.28±0.23vs 4.64±0.35 vs 6.37±0.36, p<0.05), compared to the other three groups. Additionally, these imaging findings correlated well with the histological confirmation.

**Conclusion:**

It is feasible to use RFH to enhance HSV-TK/GCV gene therapy of hepatic tumors in *in-vitro* and *in-vivo* settings, as assessed by molecular imaging. This technical development may provide a novel opportunity for effective treatment of liver malignancies by employing simultaneous integration of radiofrequency technology, interventional oncology, and direct intratumoral gene therapy.

## INTRODUCTION

Hepatocellular carcinoma (HCC) is one of the most common malignancies worldwide, with a high mortality rate [1]. During the past 15 years, the incidence of HCC has doubled [2]. With the development of advanced imaging technology and the rising awareness of HCC risk factors, an increasing number of patients with HCC are being diagnosed at a relatively early stage [3]. The most curative treatment methods for HCC include surgical resection and liver transplantation. However, due to the high risk of surgery in patients with liver disease and limited availability of donor organs, the surgical options are often not available to the patients.

Radiofrequency ablation(RFA) has been recognized as an effective alternative to surgical resection, and has been widely used by interventional radiologists and hepatobiliary oncologists for management of early HCC [4]. RFA is performed by inserting a radiofrequency electrode to the center of a hepatic tumor, which delivers a lethal dose of thermal energy to kill tumor cells [5]. Even though RFA is highly effective in eradicating small HCCs, there is a high incidence of tumor recurrence at the margin of an ablated tumor, especially for larger tumors (> 5 cm) and tumors adjacent to large vessels (i.e. “heat sink effect”) [6–8]. Furthermore, sublethal thermal energy in the margin of tumors from an incomplete RFA can promote cell proliferation, tumor progression, invasion, metastasis, angiogenesis, and immune response [9], decreasing the efficacy of the RFA treatment.

Efforts have been made to address this critical and challenging clinical issue by combining RFA with a variety of other treatment methods, such as transarterial chemoembolization and systemic chemotherapy [10]. However, most HCCs are resistant to chemotherapeutic agents [11–13]. Therefore, it is essential to explore other alternatives methods for effective treatment of HCCs.

Gene therapy is apromising treatment option for tackling various difficult-to-treat diseases, including cancer. So far, more than 1600 clinical trials have been conducted worldwide, with two-thirds of them on cancer treatment. Among the various oncologic gene therapies, the most widely accepted one is tumor suicide gene therapy, i.e. herpes simplex virus-thymidine kinase (HSV-TK)/ganciclovir (GCV) therapeutic system [14, 15]. HSV-TK gene is transduced to tumor cells, which then induces the expression of HSV-TK to convert the non-toxic prodrug, GCV, to its toxic metabolites, thereby interrupting the process of DNA replication and leading to cell death [16]. However, HSV-TK/GCV-mediated gene therapy has not yet been widely accepted in clinical practice for treatment of HCCs, largely due to the low efficiency of gene delivery to tumor tissues via systemic administration approaches.

Recent studies have demonstrated that mild hyperthermia (at 42°C) can enhance gene transfection efficiency and gene therapeutic effect on various tumors [17–19]. The recognized mechanisms of RFH-enhanced gene therapies include tissue fracturing by heating, increased permeability of the cytoplasmic membrane, amplified cellular metabolism, and activation of the heat shock protein pathway [20]. These mechanisms facilitate the entrance of therapeutics into the target tumor cells, thereby promoting effective destruction of tumors. These exciting results led to the present study, in which we attempted to evaluate the synergistic effect of HSV-TK/GCV-mediated tumor suicide gene therapy and RFH *in-vitro* and rat models of HCC that can be monitored using multimodality molecular imaging.

## RESULTS

### *In-vitro* experiments: RFH-enhanced HSV-TK/GFP gene expression and killing effect on hepatic cancer cells

**Figure 1:**
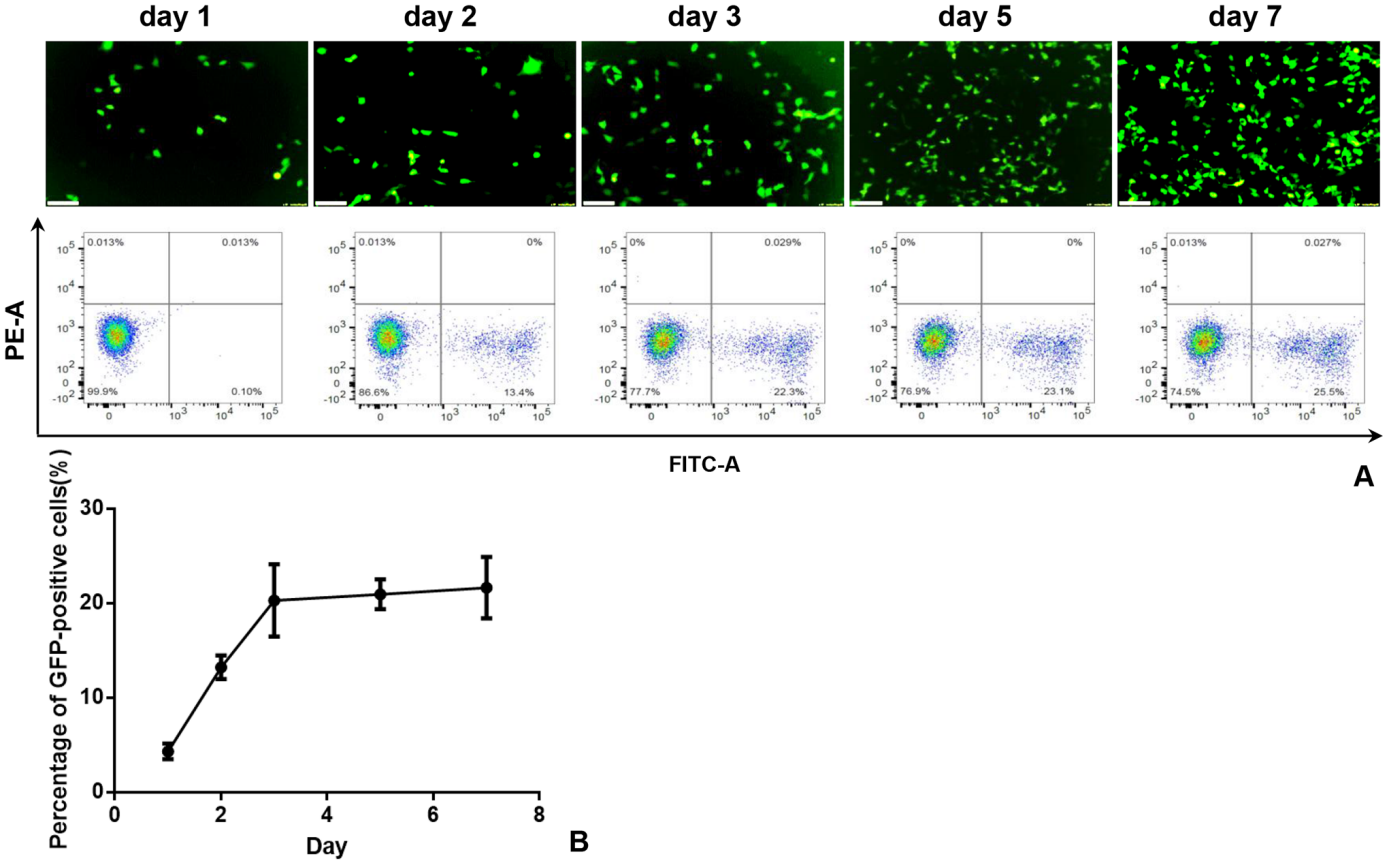
**(A)** Fluorescent microscopy (upper panel) and flow cytometry analysis (lower panel) show the GFP gene expression in cells reaches the peak at day 3 and maintains the steady level from day 3 to day 7 post-transduction **(B)**.

**Figure 2:**
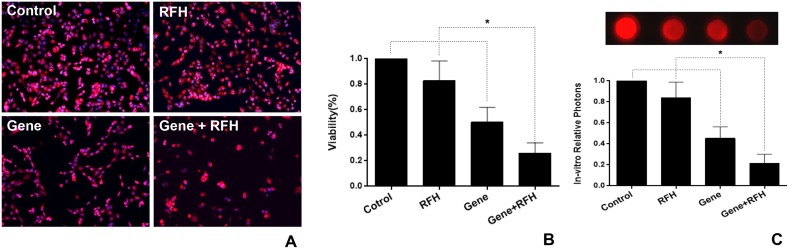
**(A)** Fluorescent microscopy demonstrated fewer survived cells in the combination therapy group than the cells in the groups treated with gene therapy alone, RFH alone and PBS. **(B)** MTS assay further confirmed the lowest cell viability in combination therapy, compared to the other treatments (^*^=p<0.01). **(C)** *In-vitro* bioluminescence optical imaging shows the lowest photon signal intensity in the combination therapy group of gene therapy with RFH (^*^=p<0.01).

**Figure 3:**
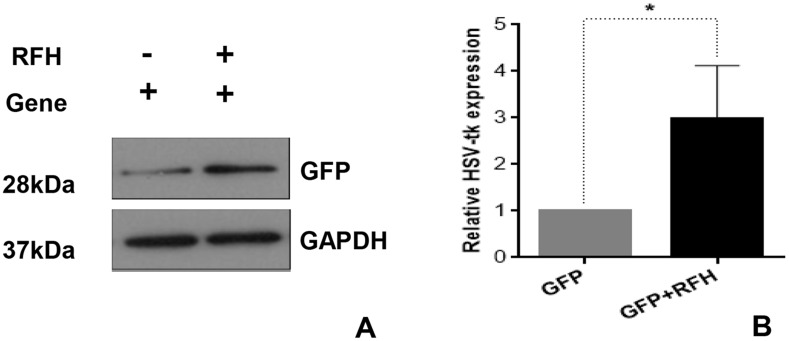
Western blotting analysis shows that GFP expression is significantly higher in the cells group with RFH enhancement, compared to the cells without RFH enhancement (^*^p=0. 0015).

Flow cytometry demonstrated that the expression of HSV-TK/GFP gene reached the peak at day 3 after the gene transfection, and then maintained at the same level from day 3 to 7 (Figure 1). Thus, we selected day 3 post-gene transfection as the time point of assessing killing effects on cells. Confocal microscopy demonstrated fewer survived cells in the combination therapy group (Gene + RFH) compared to the groups treated with gene therapy alone, RFH alone, and PBS (Figure 2A). This finding was further confirmed by quantitative MTS assay, which showed the lowest cell proliferation with the combination therapy, compared to the three other treatments (26.1±3.2% vs 50.4±4.6% vs 82.9±6.3% vs 100%, p<0.01) (Figure 2B). The bioluminescence assay of cells also displayed a significantly lower level of relative photon signal intensity in the combination therapy group, compared to othergroups (24.0±2.7% vs 50.1±2.3% vs 74.2±9.0% vs 100%, p<0.01) (Figure 2C). Compared to gene transfection alone, gene transfection in combination with RFH demonstrated an elevated expression level of HSV-TK gene, indicating that RFH up-regulated the expression of HSV-TK (Figure 3).

### *In-vivo* experiments: RFH-enhanced HSV-TK/GFP gene therapy on rat orthotopic hepatic tumors

**Figure 4:**
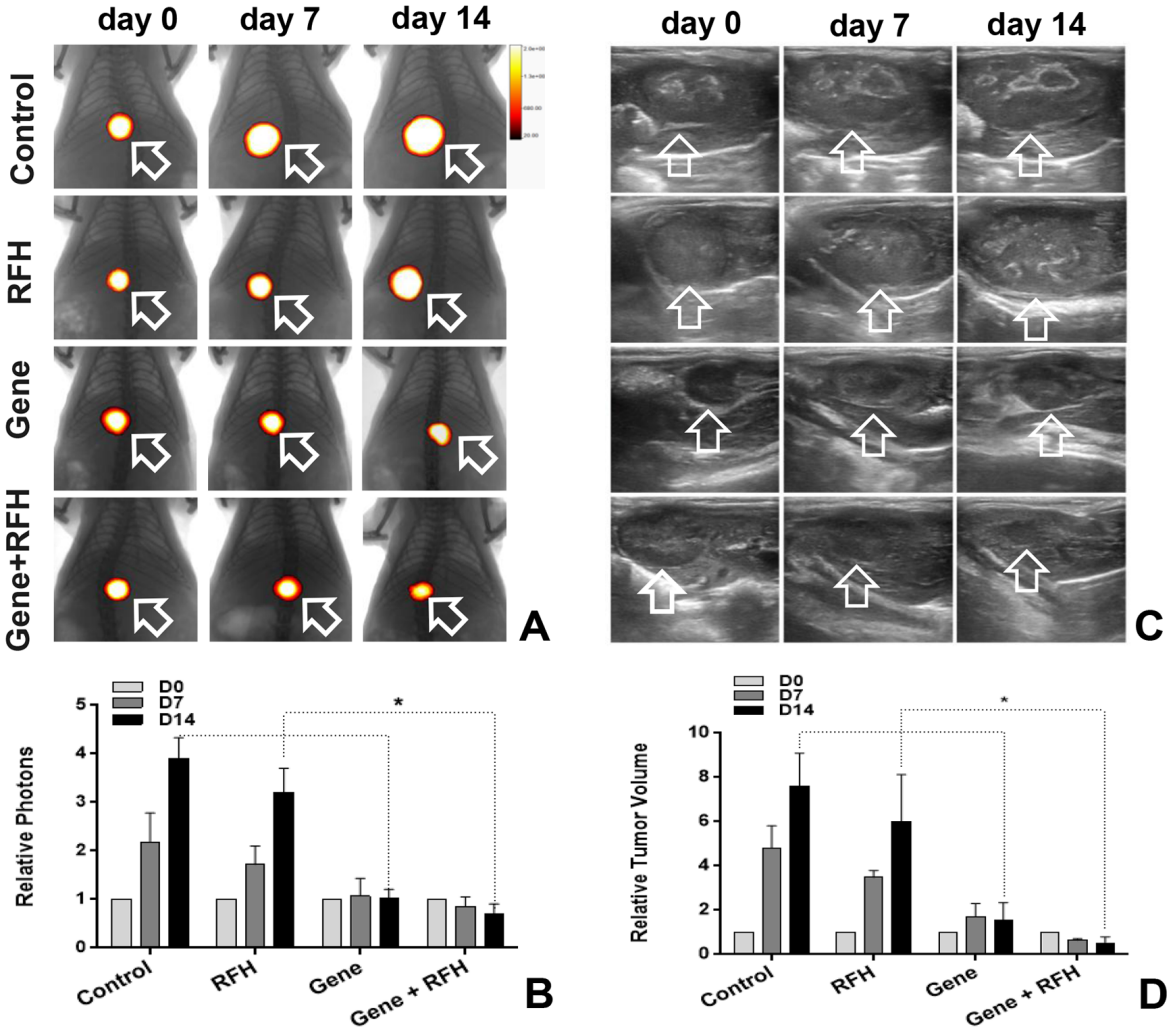
**(A** and **B)** Optical/x-ray imaging to assess the tumor growth and response to the treatments at days 7 and 14 after the treatments shows the lowest bioluminescence signals (orange-yellow color, arrows inA) in the combination therapy group, compared to the other control groups (^*^p<0. 05). **(C** and **D)** Ultrasound imaging shows the decreased tumor size (arrows) of the group with combination therapy, compared to the other three treatments (^*^p<0.05).

**Figure 5:**
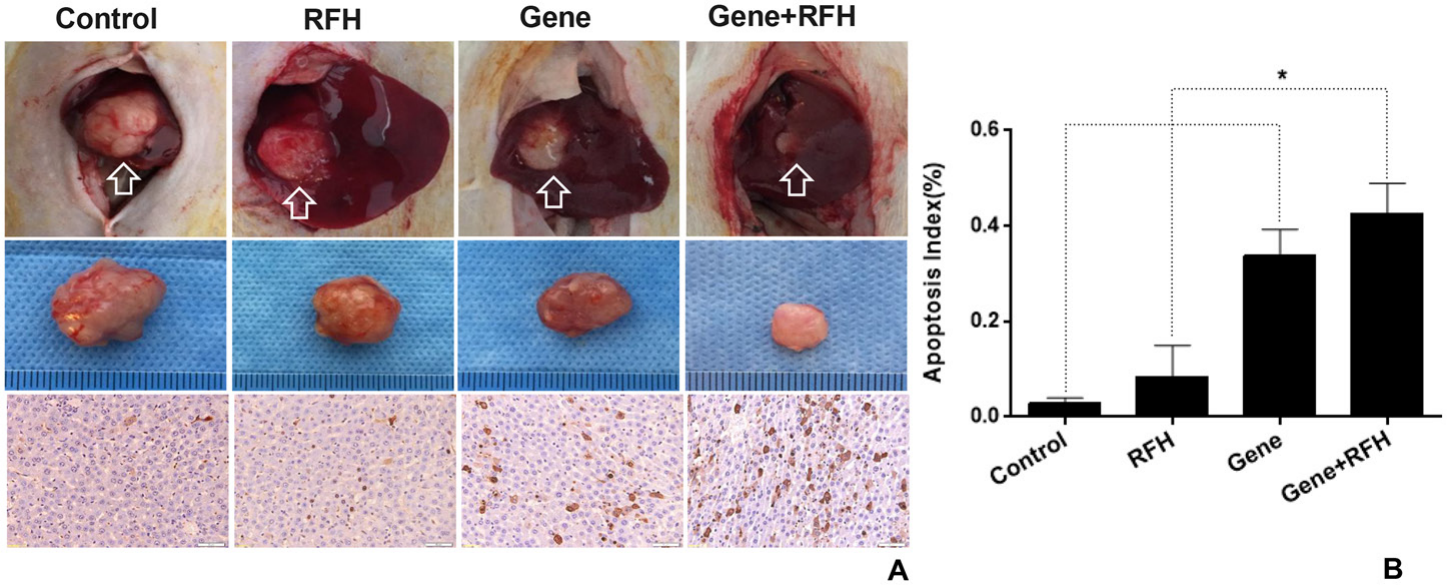
**(A)** Gross specimens of the tumors demonstrated the smallest tumor size in the group treated with combination therapy (upper and middle panel), with more apoptosis cells (brown color dots, low panel) (×20 magnification). **(B)** Apoptosis analysis by TUNEL further confirms a significantly higher apoptosis index in the combination therapy group compared to the other three groups.

**Figure 6:**
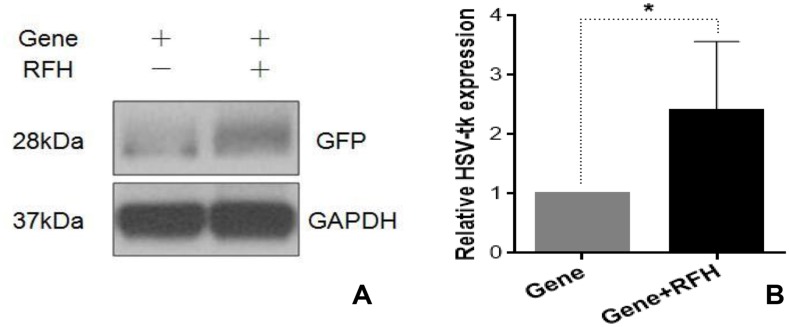
Western blotting analysis shows the higher GFP gene expression in the tumors with RFH enhancement than the tumors with HSV-TK/GFP gene transduction alone (^*^p<0. 05).

All 24 rats with orthotopic hepatic tumors survived the entire experimental procedures without complications. Bioluminescent optical imaging detected a significant decrease in relative photon signal in the combination therapy group (Figure 4A), compared to the other three groups treated with intratumoral HSV-TK/GCV alone, intratumoral RFH alone, and PBS (0.57±0.09 vs1.06±0.10 vs 3.43±0.27 vs 3.85±0.12, p<0.05) (Figure 4B). Ultrasound imaging also showed the smallest relative tumor volumes in the combination therapy group (Figure 4C), compared to the other three groups (0.28±0.11, 1.28±0.23 vs 4.64±0.35 vs 6.37±0.36, p<0.05) (Figure 4D). Gross specimens obtained 2 weeks after each treatment displayed the smallest tumor size in the combination therapy group, compared with the other groups (Figure 5). Apoptosis analysis with TUNEL further confirmed a significant increase in apoptotic cells with the combination therapy, compared to the other three treatments (2.89±0.44% vs 8.53±2.64% vs 33.8±2.23% vs 44.26±2.58%) (Figure 5). Western blot analysis confirmed that the expression of GFP gene in tumor tissues was approximately two-fold higher in the group treated with gene transfection combined with RFH than in the group treated with gene transfection alone (Figure 6).

## DISCUSSION

Image-guided RFA has been used as one of the principle modalities for treatmentof HCCs smaller than 5 cm. However, inadequate or sublethal ablation may actually accelerate the malignant cell transformation and tumor progression [21], contributing to recurrence especially for the tumors larger than 5cm [22, 23]. In order to eradicate a tumor by thermal ablation, complete negative margin needs to be accomplished. However, in clinical practice, this is often not technically feasible due to some intrinsic limitations of RFA, such as the “heat sink” effect caused by adjacent large vessels, and the proximity to normal vital structures including central bile ducts and hollow viscus. It is essential to explore alternative strategies to solve this clinically important problem and to maximize the curative potential of RFA in the treatment of HCC.

Previous studies from our group and other groups have confirmed that mild hyperthermia (42-44°C) can enhance the therapeutic effect of chemotherapy of HCCs [24]. However, both primary and metastatic hepatic malignancies can develop resistance to chemotherapy, primarily caused by tumor-initiating cells [25]. Thus, in this study, we attempted to explore an alternative to chemotherapy and studied the feasibility of using intratumoral HSV-TK/GCV gene therapy in conjunction with RFH in rat models with orthotopic HCC that can be assessed using molecular imaging techniques.

Gene therapy represents one of the most exciting frontiers in modern medicine. Selecting an efficient approach to increase gene transfection and expression in target tumor cells is essential in developing a successful gene therapy. Gene transfection and expression depend on the utilization of suitable gene-carrying vectors. Adenoviral vectors can achieve a high level of transgene expression in tumor cells [26]. However, the relatively high risk of immune reaction caused by adenoviral vectors may prevent its routine use in human clinical applications [27]. Adeno-associated viral (AAV) vectors have a relatively high safety profile but with lower gene transfection efficiency [28], especially in HCC cells [29]. Lentivirus vectors can transduce tumor cells more efficiently than AAV vectors [30] and cause less immune sensitization than adenoviral vectors. One study demonstrated that lentiviral vectors can efficiently transduce HCC cells while sparing normal hepatocytes [31]. Our study showed that RFH could enhance HSV-TKgene expression in both rat hepatic cancer cells and rat orthotopic hepatic tumors.

In the present study, we first established a rat model with orthotopic rathepatic cancer cells that can be detected by optical imaging. Compared to the mouse model with subcutaneous HCC xenografts, our rat orthotopic hepatic cancer model should more closely mimic the pathophysiologic properties of human HCC. By using this model, we confirmed that RFH could significantly enhance the therapeutic effect of HSV-TK/GCV on hepatic tumors, as demonstrated by decreased cancer cell viability, reduced bioluminescent signal intensities, and decreased tumor volumes in the group treated with HSV-TK gene therapy and RFH, in comparison to RFH or gene therapy alone.

In our study, we delivered a high dose of HSV-TK/lentivirus intratumorally through the multiple infusion needles of the multipolar RF electrode which were deployed under ultrasound guidance to ensure the full coverage of the tumor margin. This permitted us to generate sublethal hyperthermia at the margin of the ablated tumor to enhance the tumoricidal effect of HSV-TK therapy.

To prove the principle of the new technique, we set the hyperthermia temperature at 42.0°C primarily tumoricidal to achieve gene therapy enhancement. Future series of studies are warranted to optimize the temperature and to maximize the enhancing effect of RF hyperthermia on gene therapy. In addition, we had to limit our follow-up time for the animal study to two weeks after the treatment. A longer follow-up period would have resulted in the excessive growth of hepatic tumors in the control animal group, which was not allowed by our Institutional Animal Care and Use Committee.

In summary, our study showed that it is feasible to use interventional RFH to enhance intratumoral HSV-TK/GCV-mediated gene therapy onrat orthotopic HCCs. This study integrates various technical and clinical aspects of modern medicine, including RF technology, interventional oncology, and direct intratumoral gene therapy. The demonstrated potential of the combination RFH and gene therapy offers a novel opportunity to improve cure rates for HCC.

## MATERIALS AND METHODS

### Study design

The study included two parts: (a) *in-vitro* experiment to establish the “proof of principle” that RFH could enhance HSV-TK gene expression in hepatic cancer cells by analyzing GFP/HSV-TK gene expression in rat hepatic cells using western bolting, evaluating the viabilities using MTT and bioluminescence signal of treated cells optical imaging in each group; and (b) *in-vivo* experiment to validate the technical feasibility of using molecular optical and ultrasound imaging to monitor RFH-enhanced HSV-TK/GCV gene therapy in rat models with orthotopic hepatic tumors. Rat orthotopic hepatic cancers were treated with intratumoral HSV-TK gene therapy alone, RFH alone, RFH+HSV-TK gene therapy and PBS as a control. The tumor size and bioluminescence signal were measured and correlated with pathology.

### *In-vitro* experiments

#### Production of HSV-TK - green fluorescence protein (GFP) gene/lentivirus

LentiFectin^™^ transfection reagent(G074), pLenti-III-HSVTK(LV047), and third generation (LV053) packaging mix were purchased from Applied Biological Materials (ABM) Inc. (Richmond, Canada). The pLenti-III-HSVTK plasmids were amplified, extracted, and purified using the Qiagen Hi speed plasmid max kit (Qiagen, Inc.Valencia, CA, USA). All experiments of producing HSV-TK-GFP/lentiviral particles were performed according to the protocols provided by the manufacturers. Viral titer was detected using the method as described in the literature [32].

#### RFH-enhanced HSV-TK/GCV killing efficacy on HCC cells

Rat HCC cells (McA-RH7777, American Type Culture Collection, Manassas, VA, USA) were maintained with DMEM medium (Hyclone, South Logan, UT, USA) supplemented with 10% fetal bovine serum (Thermo Fisher Scientific Inc, Waltham, MA, USA) at 37°C in a humidified atmosphere containing 5% CO2. The cells were first transfected with luciferase (Luc)/red fluorescence protein (RFP) gene/lentivirus, to produce Luc/RFP-positive cells according to the manufacturer's protocol (GeneCopoeia Inc., Rockville, MD, USA). Luc/RFP-positive cells were sorted out using fluorescence-activated cell sorting technique (Aria II, Becton Dickinson, Franklin Lakes, NJ, USA), and then cultured in DMEM medium supplemented with 10% fetal bovine serumat 37°C in a humidified atmosphere containing 5% CO_2_.

1×10^5^Luc/RFP-cells were seeded in four-chamber cell culture slides (Nalge Nunc International, Rochester, NY, USA). Cells in different groups were treated by (a) HSV-TK/lentivirus gene therapy at a MOI of 20/well combined with 30 minutes of RFH at approximately 42°C, followed by 72 hours of GCV exposure (100mmol/L); (b) HSV-TK/lentivirus gene therapy followed by GCV exposure alone; (c) 30-min RFH alone; (d) phosphate buffered saline (PBS) to serve as a control. The lentiviral transduction was performed according to the manufacturers’ instructions, and RFH was performed as described in the literature [17, 33, 34].

### Cell viability assay

Cell viability was evaluated by MTS assay 72 hours after GCV treatment. 10-μl MTS reagent was added into each cell culture well, and then cells were incubated at 37 °C for 4 hours. The absorbance was detected at 490 nm with a Microplate Reader (VersaMax; Molecular Devices, Sunnyvale, CA, USA). The relative cell viability of each group was evaluated using the equation A_treated_-A_blank_ / A_control_-A_blank_, where A is the absorbance. Cells on slides were subsequently washed twice with PBS, fixed in 4% paraformaldehyde, and then counter stained with 4', 6-diamidino-2-phenylindole (DAPI, Vector Laboratories, Burlingame, CA, USA). The slides were imaged with a laser confocal microscope (A1R; Nikon, Tokyo, Japan). The experiments were repeated six times for each group.

### Cell bioluminescence assay

Luc/RFP-cells were suspended in 50-μl cell culture medium. After 5-μL Pierce D-Luciferin (ThermoFisher Scientific, Rockford, IL, USA) was added into the cell suspension, the cells were incubated for 20 minutes. Cell suspension was mixed with 50-μl Matrigel (CORNING, Bedford, MA, USA) in a cylindrical glass tube to detect the bioluminescence using an *In Vivo* Optical Imaging System (Bruker Corp, Billerica, MA, USA). The signal intensity of bioluminescence emitting from cells was quantified as the mean of all detected photon counts within a manually placed region of interest (ROI) using the Burker MI software. Data was normalized to relative signal intensity (RSI) according to the following equation: RSI = SI_T_/SI_C_, where SI is signal intensity, T is the treatment group, and C is the control group.

### Western blotting

Expression of HSV-TK/GFP gene in different groups was quantified by western blotting three days after gene transfection. Cell lysate was prepared from cultured cells from the different groups. 50-μg of lysates were separated by 10% SDS-PAGE and then electro-transferred to a nitrocellulose membrane. After being blocked in blocking buffer (5% non-fat milk in tris-buffered saline with 0.05% Tween 20) for 1 hour, each nitrocellulose membrane was incubated with various primary antibodies overnight at 4°C. Antigen-antibody complexes were then visualized using enhanced chemiluminescence reagents. All antibodies were purchased from Thermo Fisher (primary antibody, MouseMonoclonal antibody 1:3000; secondary antibody, Rabbit anti-ratantibody, 1:5000. Thermo Fisher Scientific Inc, Waltham, MA, USA.). The level of gene expression was quantified by measuring the density of the immunoreactive bands and subsequently normalizing to relative areas (RA) according to the following equation: RA = A_RFH+gene_/A_gene_, where A represents the chemiluminescent area of the different groups.

### *In-vivo* experiments

#### Animal model with orthotopic hepatic cancer

The animal experiments were approved by our institutional Animal Care and Use Committee. Orthotopic hepatic tumors were created in nude rats (RNU Rat, Charles River, Skokie, IL, USA). Under anesthesia with 1–3% isoflurane (Piramal Healthcare, Andhra Pradesh, India) in 100% oxygen and using an aseptic technique, the rat liver was exposed through a subxiphoid abdominal incision, and then Luc/RFP-positive McA-RH7777 cells (0.5-1×10^7^) were inoculated into the left lobe of the rat liver. The abdominal wall was closed in layers using absorbable sutures. Ultrasound imaging was used to moniter the size of each tumor.

#### RFH-enhanced gene transfection

**Figure 7:**
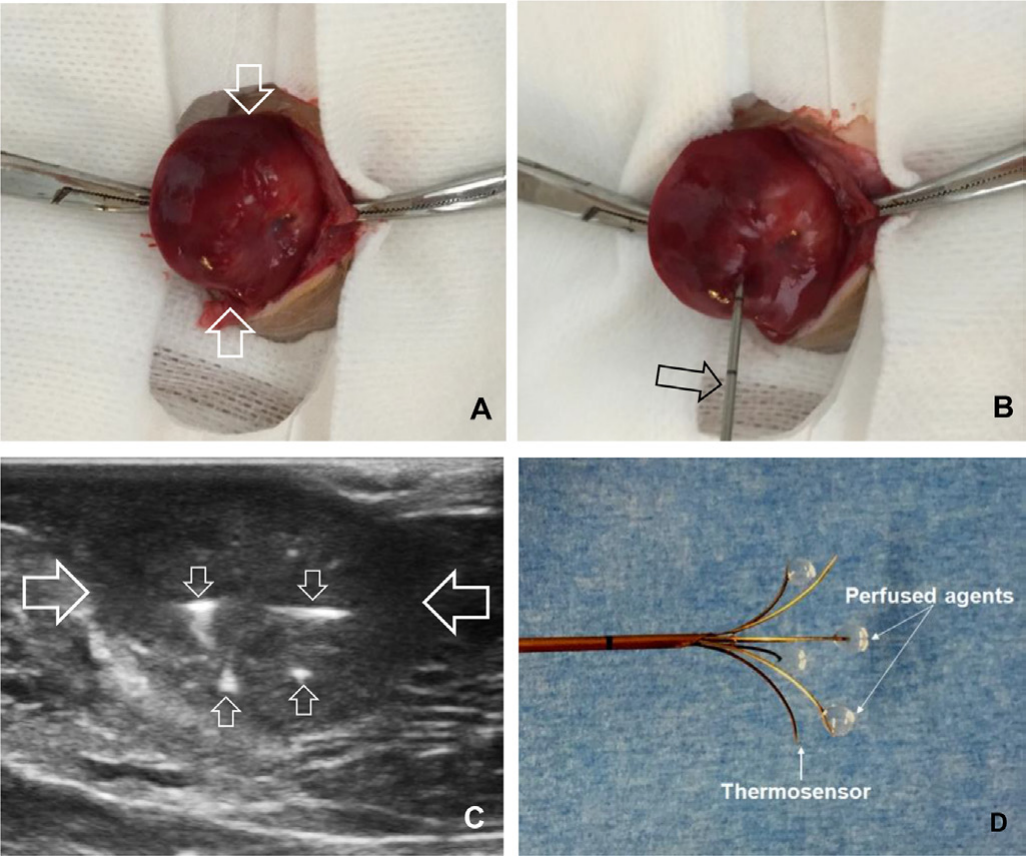
**(A)** An orthotopic hepatic tumor (arrows) is created in the rat liver. **(B-D)** Under real-time ultrasound imaging guidance, the electrode is precisely placed into the tumor (between large arrows) and then multiple infusion needles (small arrows) of the electrode is opened to deliver HSV-TK/lentivirus, followed by intratumoral RF heating using the same electrode.

Twenty-four rats with 5-10 mm in diameter were enrolled in the treatment experiments and were divided into four treatment groups: (a) direct intratumoral injections of 1×10^8^pfu HSV-TK lentiviral particles in 100-μL PBS using a multipolar RF electrode, immediately followed by intratumoral RF heating at approximately 42°C for 30 minutes; (b) intratumoral injection of 10^8^HSV-TKpfu lentiviral particles only; (c) 30-min RFH only; and (d) intratumoral injection of 100-μL PBS to serve as a control. The intratumoral RFH with gene injection was performed by precisely deploying the RF electrodein the tumor under real-time ultrasound imaging guidance (Figure 7). After confirming the appropriate deployment of the RF electrode within the tumor, the electrode was connected to the RF generator (Welfaremedic, Beijing, China) to deliver thermal energy to the tumor sat approximately 42°C for 30 minutes.

#### Optical and ultrasound imaging to follow up thetumor growth

Optical imaging (Bruker *In-Vivo* Xtreme Imaging Systems, Bruker Corp., Villerica, MA, USA) was performed to evaluate the tumor responses to gene therapy, 20 minutes after intraperitoneal injection of Pierce D-Luciferin at 150 mg/Kg (ThermoFisher Scientific, Inc, Waltham, MA, USA). Each animal was imaged at day 0 before the treatment, and at days 7 and 14 after the treatment. The intensity of bioluminescence signal of each tumor was quantified as the sum of all detected photon counts using the Bruker MI software (*In Vivo* Imaging System: Bruker *In-Vivo* Xtreme Imaging Systems, Bruker Corp., Villerica, MA, USA). The data were normalized to relative signal intensity (RSI) according to the equation:RSI = SI_Dn_/SI_D0_, where SI is signal intensity, Dn is days after treatment, and D0 is the day before treatment.

Ultrasound imaging(Sonosite Inc, Bothell, WA, USA) was then used to evaluate the tumor size changes at days of7, and 14 after the treatment. The axial (X) and longitudinal (Y) diameters of tumors, as well as the tumor depths (Z) were measured on ultrasound images to assess the maximal dimensions. The volume was calculated using the following equation:volume= X^*^Y^*^Z^*^π/6, which was then normalized to relative tumor volume (RTV) according to the equation: RTV = V_Dn_/V_D0_, where V is tumor volume, Dn is the day after the treatment, and D0 is the day before the treatment.

#### Histologic correlation/confirmation

Tumors were harvested, fixed in 10% formaldehyde, embedded in paraffin, and sliced at thickness of 4-8μm. Apoptotic cells were detected by terminal deoxynucleotidyl transferase dUTP nick end labeling(TUNEL), using a cell apoptosis detection kit(Roche Molecular Biochemicals, Pleasanton, California). Six different fields were randomly selected to calculate the percentage of TUNEL stain positive cells on each slide using an Olympus DP72 digital camera (Olympus, Tokyo, Japan) and to calculate apoptotic index(AI), where AI = (number of apoptotic cells/the total number of tumor cells)×100%.

### Western blotting

A portion of the harvested tumor tissues was homogenated and digested. Expression of HSV-TK/GFP gene in tumor tissues was quantified with western blotting using the protocol described above.

### Statistical analysis

Statistical analysis was performed using the statistical software (SPSS, Version 19.0; Chicago, IL, USA). Non-parametric Mann-Whitney U test was used to compare (i) relative proliferation rates and bioluminescent signal intensities among different cell groups; (ii) relative bioluminescence signal intensities of tumors; and (iii) relative tumor volumes at different time points among different animal groups. A *P* value of less than 0.05 (*P*<0.05) was considered statistically significant.
